# Surgical Treatment of Iatrogenic Rectourinary Fistula—York-Mason Technique—a Case Report

**DOI:** 10.5402/2011/292517

**Published:** 2011-06-15

**Authors:** Pedro Bargão Santos, Fernando Ferrito, Rocha Pires

**Affiliations:** ^1^Department of Urology, Hospital Prof. Doutor Fernando Fonseca, EPE, IC 19, 2720-276 Amadora, Portugal; ^2^Department of General Surgery, Hospital Prof. Doutor Fernando Fonseca, EPE, IC 19, 2720-276, Portugal

## Abstract

*Introduction*. Recto-urinary fistulas resulting from trauma or surgery are a serious and debilitating complication. They represent a challenge not only because of the difficulty on choosing the best technique to solve them but also because of the risk of recurrence. Spontaneous cure is rare. *Materials and Methods*. We describe the case of a 61-years-old man that on the 9th postoperative day of a laparoscopic radical prostatectomy (LRP) started with fecaluria and liquid faeces. Recto-urinary fistula was confirmed at the 10th postoperative day by CT scan and contrast enema. *Discussion*. We chose the *York-Mason* technique, because it is simple to perform, effective and has minimal morbidity. This is a posterior, transrectal, and transsphincteric approach, carried out on healthy tissues without previous scarring phenomena. *Results*. The postoperative period progressed without complications, and the patient discharged on the 4th day. The closure of the fistula was confirmed radiologically by retrograde cystography after 4 weeks allowing the removal of drainage catheter. The reconstruction of intestinal transit was carried out 2 months later. *Conclusion*. The *York-Mason* technique, a transrectal and transsphincteric approach with minimal morbidity, proved to be effective on the resolution of the recto-urinary fistula, a rare complication of the radical prostatectomy.

## 1. Introduction

Rectourinary fistulas resulting from trauma or surgery are a serious and debilitating complication of genitourinary surgery because of its rarity and due to the poor surgical experience of the urologist in its correction. The corrective surgery is therefore a challenge not only for choosing the best technique as for the fear of recurrence. Spontaneous cure is rare.

## 2. Materials and Methods

We describe the case of a 61-years-old man that on the 6th day after a laparoscopic radical prostatectomy (LRP) initiates nausea, vomiting, and fever. On the 9th day he becomes with fecaluria and liquid stool. The fistula was confirmed on day 10 after the realization of abdominal and pelvic CT and contrast enema. We proceeded to an infraumbilical laparotomy for the removal of clots, correction of vesicourethral anastomosis and direct attempted of closure of the injury of the rectum wall. Although we had completed the surgery with a colostomy, the fistula remained. From the different ways of approach, we chose the York-Mason technique because it is simple to perform, effective, and has minimal morbidity.

**Figure 1:**
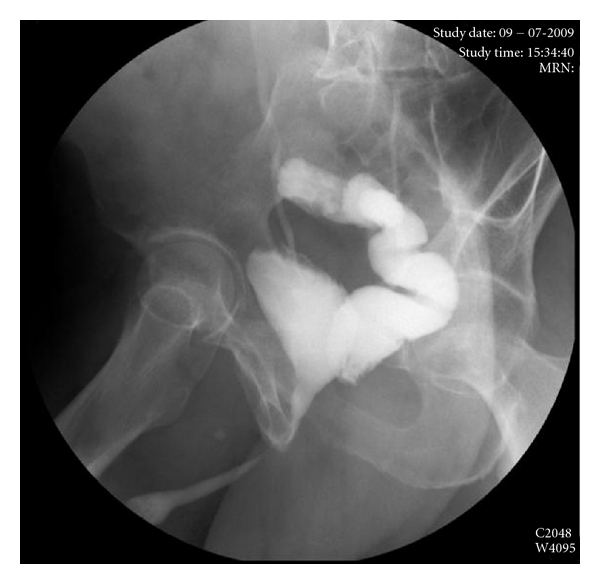
Rectourinary fistula after LRP. The retrograde cystourethrography shows the filling of the bladder and the contrast in the rectum.

The preoperative evaluation included, in addition to routine examinations, cystoscopy to visualize the fistula and its relationship with the surrounding structures, including the urethral meatus being the fistulous orifice in the midline above the bladder trigone. The digital rectal examination revealed no alterations, and the retrograde cystourethrography showed the contrast passing to the rectum (Figure 1). The colon was prepared according to the protocol of the service.

**Figure 2:**
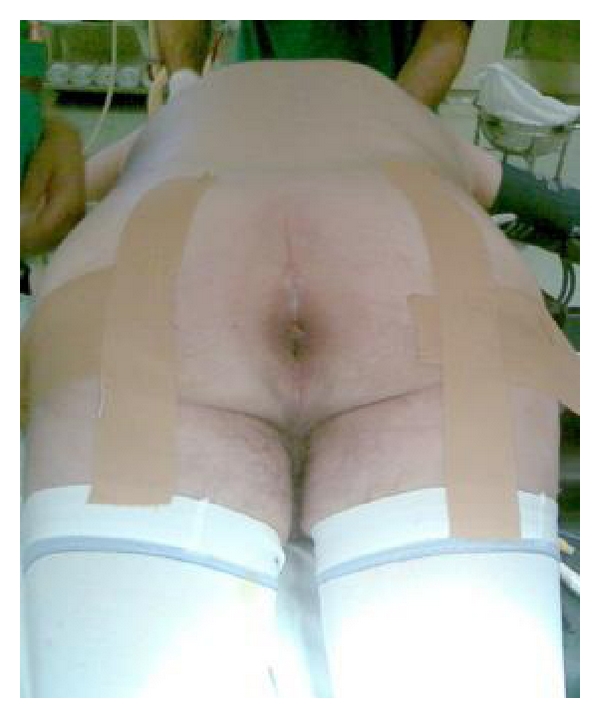
Prone position—*Jack-Knife* position.

In the operating room, the procedure begins by refering the fistulous track by cystoscopy with a 6th Fr catheter, tied to a 18th bladder catheter. The patient is placed in prone position—*Jack-Knife* position—and buttocks kept away with adhesive (Figure 2).

**Figure 3:**
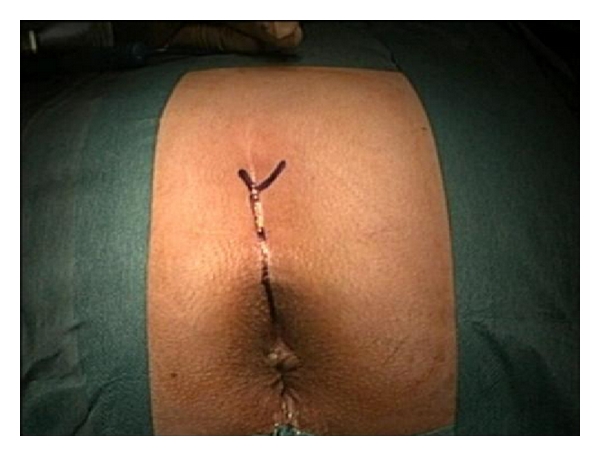
The surgical incision of York—Mason technique—starting at the right side of the coccyx towards the anal edge by the midline.

**Figure 4:**
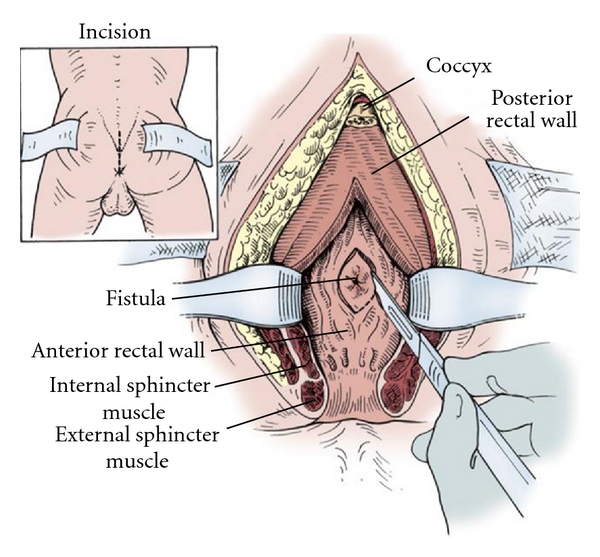
Diagram of the York-Mason technique (from Middleton RG: rectourethral fistula repair. In Krane RJ, Siroky MB, Fitzpatrick JM, eds: Operative Urology. Philadelphia, Churchill-Livingstone, 2000:286).

**Figure 5:**
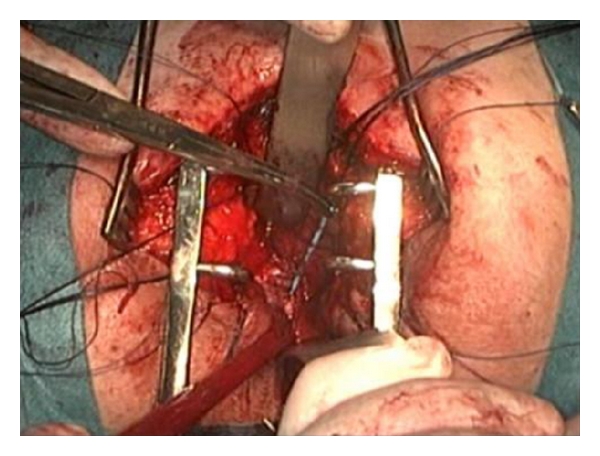
Dissection and identification of the fistulous track, respectively.

**Figure 6:**
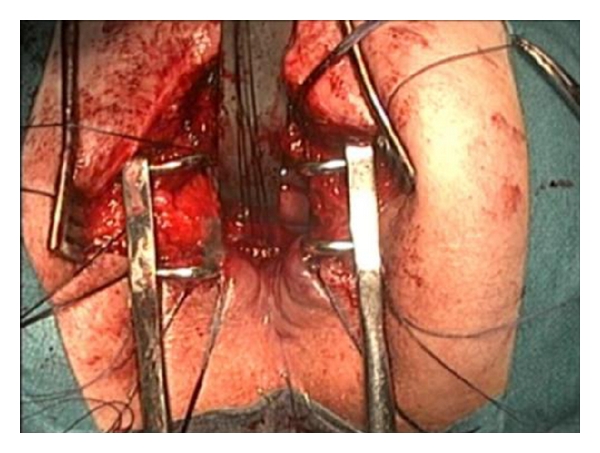
Dissection and identification of the fistulous track, respectively.

The incision starts at the right edge of the coccyx and extends to the midline to the anal edge [1–3] (Figure 3). Continues to the subcutaneous tissue, carefully dividing the muscle layers of the sphincter and identifying them with an absorbable 000 suture. It is essential to the thoroughness of this step to ensure a good approximation, realignment, and reconstruction of the sphincter. After the incision of the posterior wall of the rectum, the anterior rectum wall, and the fistulous orifice become visible which in turn is referred with 2 suture lines. The fistulous track is isolated with the scalpel blade and the scissors creating the planes of dissection between the wall of the rectum and the urinary tract, allowing good plans for closure without tension and with healthy edges. In this case, we did not do the excision of the fistulous track because of the proximity of the ureteral meatus (Figures 4, 5, and 6). The posterior wall of the rectum is closed with an absorbable 00 suture, and the reconstruction of the muscle layers of the anal sphincter previously referred is done. A subfascial drain remains for 48 hours.

## 3. Results

**Figure 7:**
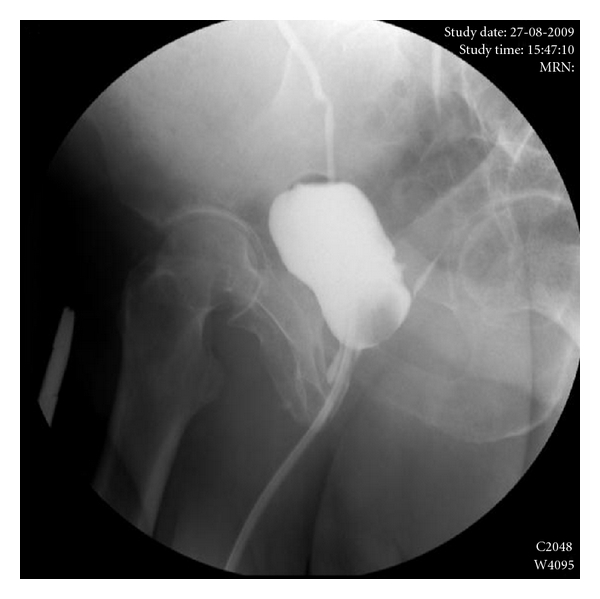
Cystography performed 4 weeks after surgical correction of rectourinary fistula. It is possible to observe complete absence of fistula.

The entire surgical procedure took about 180 minutes, and the blood loss was minimal (<150 mL). The patient was discharged the hospital after 4 days, without any postoperative morbidity, as infection, abscess, or pain. The bladder catheter was removed at week 4 after cystography (Figure 7). The colostomy was closed after eight weeks. There was no stenosis or fecal incontinence. The patient is currently in a rehabilitation program for stress urinary incontinence.

## 4. Discussion

Rectourinary fistula (RUF) is a rare complication of genito-urinary surgery. It is estimated that 60% of these fistulas are iatrogenic, occurring not only during surgery of radical prostatectomy (1-2%) [1] as well as after radiotherapy, brachytherapy, and cryotherapy [4]. The therapeutic approach of this complication is often a frustrating challenge. Not only for the urologist because of the lack of experience and the risk of recurrence of the pathology but also because of the important changes in patient quality of life since the spontaneous cure is rare, and conservative measures often involve fecal and urinary diversion [1].

The majority of the RUF must be treated surgically, although some will eventually close with conservative treatment [3, 5]. Fistulas that develop after laparoscopic radical prostatectomy or open approach may close spontaneously with bladder drainage, bowel rest, and parenteral nutrition. In some cases, fecal diversion is necessary. Rassweiler et al. [6] in 2003 described the success of conservative treatment in 6 of 8 patients with RUF, requiring temporary colostomy only in 2 patients. Noldus et al. [7] in 1999 described the closure of RUF after radical prostatectomy and radical cystectomy with conservative treatment in 7 of 13 patients. The other 6 were treated successfully with the *Latzko* procedure (transanal). The success of conservative treatment was equally represented with endoscopic suturing, fulguration of the fistula and the application of fibrin glue [8].

Surgical treatment of the FRU is challenging and the basic principles of fistula repair technique in this pathology have a special importance, namely.

1. Adequate exposure of the fistula with debridement of devitalized and ischemic tissues.
2. Removal of foreign bodies or synthetic materials in the region surrounding the fistula.
3. Careful dissection and anatomical separation of the surrounding organs.
4. Watertight closure.
5. Use of flaps well vascularized and its atraumatic handling.
6. Closing on several layers.
7. Suture without tension and without overlapping.
8. Adequate urinary drainage.
9. Prevention and treatment of infections with appropriate use of antibiotics.
10. Maintenance of hemostasis.

Several surgical approaches have been described, namely, techniques at one time or multiple surgical times. The question of the realization of fecal diversion before or after fistula correction is also controversial. Some authors advocate fecal diversion and correction of all RUF in more than one time [9]. This can be considered the standard conservative approach that in combination with an adequate bladder drainage allows the spontaneous healing of the fistula without direct manipulation of the urinary tract. The extent of morbidity and costs associated with multiple procedures favor the execution of the correction in one surgical time.

Thus, seems to be consensus that one time surgical approaches can be used in postsurgical situations, small RUF, not associated with infection, abscess, or poor bowel preparation [10]. The approaches in various surgical steps can be considered in cases of large RUF associated with radiotherapy, local or systemic uncontrolled infection, immunosuppression cases, or inadequate bowel preparation in the last operative time [3].

Transrectal approaches with or without section of the anal sphincter have been described for the surgical treatment of RUF. 

In 1969, *Kilpatrick and York—Mason* described a posterior approach, *transrectal, and transsphincteric - York-Mason technique*—in which all layers of the anorectal sphincter are divided for direct access to the fistula, located at the anterior rectal wall [2, 11]. Relatively simple to perform, this procedure is done through healthy tissues without scars or previous phenomena, with minimal morbidity and minimal blood loss. The main disadvantage of this technique is the difficulty in interposing other tissues such as peritoneum, *omentum,* or muscle tissue. Thus, the surgeon can opt for the reinforcement of the closure with synthetic sealants, including cyanoacrylate. The risk of fecal incontinence was proven to be completely unfounded if the procedures of the technique are respected.

In contrast to the transrectal transsphincteric technique, the *transanal approach* does not involve the section of the anal sphincter. The exposure of the fistula is achieved by the dilation of the anus and its fixed retraction. The *Latzko *procedure corresponds to one of these types of approach. Initially described for vesicovaginal fistulas, the fistula and the rectal mucosa are dissected in the four quadrants and then it is closed in 3 layers with the possibility of using rectal mucosal flaps [12–14]. The biggest disadvantage of this approach is the poor exposure and limited maneuverability in the surgery.

**Figure 8:**
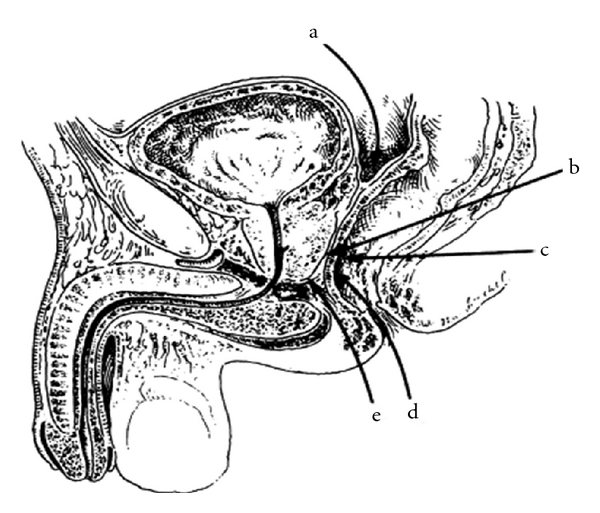
Paths of approach for the correction of rectourinary fistulas: (a) transabdominal (b) laterosacred (Kraske) (c) transsphincteric (York-Mason) (d) transanal (Latzko) (e) perineal.

The transabdominal and perineal techniques (Figure 8), most familiar to the urologist, allow the interposition of vascularized tissues however, they are usually more time consuming due to the need of surgical dissection through a territory previously handled.

The *perineal approach* has been used by some authors in selected cases. Excellent results have been demonstrated with this technique, particularly in combination with interposition of *gracilis *muscle flaps [15–17], *dartos *muscle pedicellate flaps [18], penile skin [19], levator ani muscle [20], and bladder [21].

The *transabdominal approach* has been described but with limited success [5, 9, 22]. The main advantage of this technique is the possibility of interposition of the *greater omentum*. The potential disadvantages are the increased morbidity and prolonged postoperative convalescence (associated with the laparotomy incision), the worst operative field with less maneuverability in deep pelvic space and the risk of urinary and fecal incontinence [23].

## 5. Conclusion

Rectourinary fistulas represent a major surgical challenge. The York-Mason technique [3, 23–25] allows good visualization and identification of the fistula, as well as easy access and optimal surgical exposure (see Table 1). Excellent results are demonstrated, such as the absence of anal incontinence and stricture, the absence of postoperative pain and shorter hospital stay. These results are identical in all published series. Therefore, it is considered a highly effective approach for the correction of rectourinary fistulas. 

## Figures and Tables

**Table 1:** International published series about the York-Mason technique.

References	Institutions	Number of cases	Number of successful RUF resolution
Crippa et al. [4]	University of São Paulo	7	7
Fengler and Abcarian [26]	Brooke Army Hospital University of Illinois-Chicago	8	8
Renschler and Middleton [27]	University of Utah	25	22
Stephenson and Middleton [3]	University of Utah	15	15
Kasraeian et al. [28]	Institute Mutualist Montsouris	12	12
